# Late outcomes of congenital and childhood non-immune, isolated atrioventricular block: a French nationwide retrospective cohort study

**DOI:** 10.1093/europace/euaf040

**Published:** 2025-03-11

**Authors:** Florence Mycinski, Victor Waldmann, Florence Kyndt, Béatrice Guyomarch, Alice Maltret, Marie Wilkin, Caroline Ovaert, Guy Vaksmann, Jean-Benoit Thambo, Jean-Marc Sellal, Paul Padovani, Naychi Lwin, Solène Prigent, Oscar Werner, Julien Barc, Jean-Jacques Schott, Damien Bonnet, Vincent Probst, Alban-Elouen Baruteau, The Congenital AVB study group, Florence Mycinski, Victor Waldmann, Florence Kyndt, Béatrice Guyomarch, Alice Maltret, Marie Wilkin, Caroline Ovaert, Guy Waksmann, Jean-Benoit Thambo, Solène Prigent, Claire Galoin-Bertail, Gilles Bosser, Céline Grunenwald, Grégoire De La Villeon, Bruno Lefort, Sylvie Falcon-Eicher, Matthias Lachaud, François Godart, Raphaël P Martins, Claire Dauphin, Hervé Joly, Isabelle Durand, Clément Karsenty, Guillaume Deverrière, Pierre Mauran, Bérangère Urbina-Hiel, Cécile Pascal, Adeline Basquin, Laure Ponthier, Karim Jamal-Bey, Jean-Marc Sellal, Paul Padovani, Naychi Lwin, Solène Prigent, Oscar Werner, Julien Barc, Jean-Jacques Schott, Damien Bonnet, Vincent Probst, Alban-Elouen Baruteau

**Affiliations:** Department of Pediatric Cardiology and Pediatric Cardiac Surgery, Nantes Université, CHU Nantes, FHU PreciCare, F-44000 Nantes, France; Nantes Université, CHU Nantes, CNRS, INSERM, l’Institut du Thorax, F-44000 Nantes, France; M3C-Necker, Department of Pediatric and Congenital Cardiology, Hôpital Necker-Enfants Malades, Assistance Publique-Hôpitaux de Paris, F-75000 Paris, France; Electrophysiology Unit, European Georges Pompidou Hospital, Paris, France; Adult Congenital Heart Disease Unit, European Georges Pompidou Hospital, Paris, France; Nantes Université, CHU Nantes, CNRS, INSERM, l’Institut du Thorax, F-44000 Nantes, France; European Reference Network for Rare, Low Prevalence and Complex Diseases of the Heart-ERN GUARD-Heart; CHU Nantes, Plateforme de Méthodologie et Biostatistique, F-44000 Nantes, France; Department of Congenital Heart Diseases, M3C-Marie Lannelongue, Hôpital Marie Lannelongue, Groupe Hospitalier Paris Saint Joseph, Le Plessis Robinson, France; M3C-Necker, Department of Pediatric and Congenital Cardiology, Hôpital Necker-Enfants Malades, Assistance Publique-Hôpitaux de Paris, F-75000 Paris, France; Department of Pediatric Cardiology, CHU Marseille, FHU PreciCare, Hôpital de La Timone, AP-HM, Marseille, France; Department of Pediatric Cardiology, CHU Marseille, FHU PreciCare, Hôpital de La Timone, AP-HM, Marseille, France; Marseille Medical Genetics, INSERM U1251, Aix-Marseille Université, Marseille, France; Hôpital Privé de La Louvière, Pediatric Cardiology, Lille, France; Electrophysiology and Heart Modeling Institute, IHU Liryc, Fondation Bordeaux Université, Bordeaux, France; U1045, INSERM, Centre de Recherche Cardio-Thoracique de Bordeaux, Université de Bordeaux, Bordeaux, France; Department of Pediatric Cardiology, FHU PreciCare, CHU Bordeaux, Bordeaux, France; Department of Cardiology, CHU Nancy, Université de Lorraine, Nancy, France; Department of Pediatric Cardiology and Pediatric Cardiac Surgery, Nantes Université, CHU Nantes, FHU PreciCare, F-44000 Nantes, France; Nantes Université, CHU Nantes, INSERM, CIC FEA 1413, Department of Pediatric Cardiology and Pediatric Cardiac Surgery, F-44000 Nantes, France; Department of Pediatric Cardiology and Pediatric Cardiac Surgery, Nantes Université, CHU Nantes, FHU PreciCare, F-44000 Nantes, France; Nantes Université, CHU Nantes, INSERM, CIC FEA 1413, Department of Pediatric Cardiology and Pediatric Cardiac Surgery, F-44000 Nantes, France; Department of Pediatric Cardiology and Pediatric Cardiac Surgery, Nantes Université, CHU Nantes, FHU PreciCare, F-44000 Nantes, France; Nantes Université, CHU Nantes, INSERM, CIC FEA 1413, Department of Pediatric Cardiology and Pediatric Cardiac Surgery, F-44000 Nantes, France; Department of Pediatric Cardiology and Pediatric Cardiac Surgery, Nantes Université, CHU Nantes, FHU PreciCare, F-44000 Nantes, France; Nantes Université, CHU Nantes, INSERM, CIC FEA 1413, Department of Pediatric Cardiology and Pediatric Cardiac Surgery, F-44000 Nantes, France; Nantes Université, CHU Nantes, CNRS, INSERM, l’Institut du Thorax, F-44000 Nantes, France; European Reference Network for Rare, Low Prevalence and Complex Diseases of the Heart-ERN GUARD-Heart; Nantes Université, CHU Nantes, CNRS, INSERM, l’Institut du Thorax, F-44000 Nantes, France; European Reference Network for Rare, Low Prevalence and Complex Diseases of the Heart-ERN GUARD-Heart; M3C-Necker, Department of Pediatric and Congenital Cardiology, Hôpital Necker-Enfants Malades, Assistance Publique-Hôpitaux de Paris, F-75000 Paris, France; European Reference Network for Rare, Low Prevalence and Complex Diseases of the Heart-ERN GUARD-Heart; University Paris Cité, F-75000 Paris, France; Nantes Université, CHU Nantes, CNRS, INSERM, l’Institut du Thorax, F-44000 Nantes, France; European Reference Network for Rare, Low Prevalence and Complex Diseases of the Heart-ERN GUARD-Heart; Department of Cardiology, Nantes Université, CHU Nantes, F-44000 Nantes, France; Department of Pediatric Cardiology and Pediatric Cardiac Surgery, Nantes Université, CHU Nantes, FHU PreciCare, F-44000 Nantes, France; Nantes Université, CHU Nantes, CNRS, INSERM, l’Institut du Thorax, F-44000 Nantes, France; European Reference Network for Rare, Low Prevalence and Complex Diseases of the Heart-ERN GUARD-Heart; Nantes Université, CHU Nantes, INSERM, CIC FEA 1413, Department of Pediatric Cardiology and Pediatric Cardiac Surgery, F-44000 Nantes, France; Nantes Université, INRAE, UMR 1280, PhAN, F-44000 Nantes, France

**Keywords:** Paediatric electrocardiology, Cardiac conduction disorder, Pacemaker, Clinical outcome

## Abstract

**Aims:**

The natural history of congenital or childhood non-immune, isolated atrioventricular block (AVB) is poorly defined. We aimed at clarifying its long-term outcomes.

**Methods and results:**

We retrospectively studied 385 children with isolated, non-immune AVB diagnosed from *in utero* or up to 18 years of age, at 29 French medical centres, between 1980 and 2022. Patients with structural heart disease, endomyocardial fibrosis, or maternal antibodies were excluded. Atrioventricular block was asymptomatic in 314 (81.6%) and complete in 263 (68.3%) patients at the time of diagnosis. There was progression to complete AVB in 84/122 (68.8%) patients with incomplete AVB over 12 years (7–17). A total of 286/385 patients (74.3%) received a permanent pacemaker, implanted in the first year of life in 39 (14%) and before 10 years of age in 172 (60%) children. The pacing indication was prophylactic in 203 children (71%). Genetic screening was performed in 133/385 patients (34.5%), leading to the identification of a clinically actionable variant in 11 (8.3%) patients. After a median follow-up of 10 years (5–17), no patient died or developed endomyocardial fibrosis or dilated cardiomyopathy.

**Conclusion:**

In this large nationwide study, the long-term outcome of congenital or childhood non-immune, isolated AVB was excellent. Most children required pacemaker implantation over time, albeit often as a prophylactic measure.

## Graphical Abstract

**Graphical Abstract:**
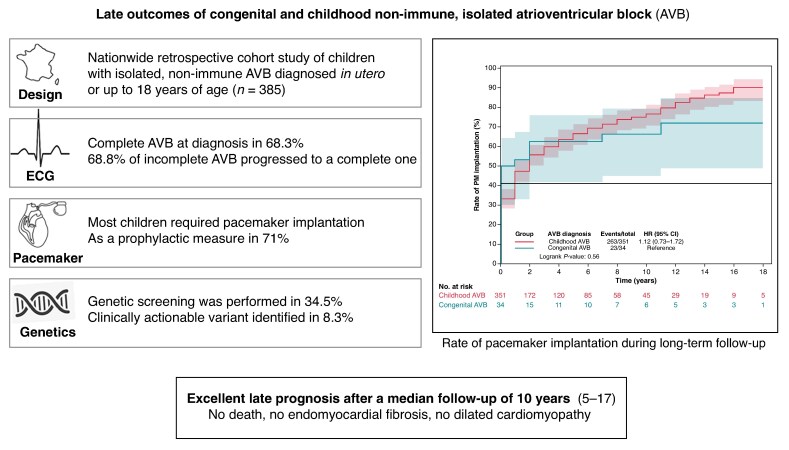

## Introduction

Immune-mediated atrioventricular block (AVB) diagnosed *in utero* or early childhood is a very rare electrocardiographic finding, associated with a risk of sudden death in the absence of cardiac pacing.^1,2^ Isolated AVBs occur in structurally normal hearts with an estimated prevalence of 1 per 15 000–20 000 live births; 90–95% of those diagnosed before 6 months of age are due to the transplacental passage of maternal anti-Ro/SSA or anti-Ro/SSA and anti-La/SSB antibodies and subsequent inflammation and fibrosis of the atrioventricular node leading to irreversible complete AVB.^3–5^ Very rarely, AVB of unknown aetiology may be diagnosed during childhood, in the absence of maternal antibodies, congenital heart disease, myocarditis, neuromuscular disorder, or any other overt cause. Long-term outcomes in these young patients with non-immune, isolated AVB are unclear, as contradictory results have been reported from large series.^6,7^ We herein aimed at clarifying late outcomes associated with this rare form of AVB.

## Methods

### Study design

A multicentre, nationwide, retrospective cohort study was conducted in 29 French tertiary hospitals from January 1980 to December 2022. Institutional review board was obtained, and all patients or legal guardians granted their informed consent to be included in the database. Patients were identified using the diagnosis database of the French hospitals’ national coding system. All patients <18 years of age diagnosed with an isolated (i.e. structurally normal heart), non-immune AVB of any type and of unknown aetiology, were eligible for the study. Patients with positive maternal anti-Ro/SSA and/or anti-La/SSB antibodies, endomyocardial fibrosis, congenital heart disease, post-operative heart block, myocarditis, neuromuscular disorder, metabolic disease, or pharmacotherapy that the mother or child was following that may have contributed to the conduction abnormalities were excluded. Patients without a baseline electrocardiogram (ECG) and/or with an unknown maternal serology were excluded from the analysis (see Supplementary methods).

### Clinical investigations and follow-up

In all patients, demographic data, personal and family history, mode of presentation, ECGs, echocardiography, treatment, and major cardiac events throughout follow-up were ascertained. In the case of device implantation, pacemaker type and mode of pacing were noted as well as device-related complications. All children underwent at least one ambulatory follow-up visit per year with a paediatric cardiologist for surveillance of their clinical status, ECG, and echocardiography. Standard 12-lead ECGs were recorded in all patients at both times of diagnosis and pacemaker implantation, or at last follow-up in non-paced patients. All ECGs were analysed by two blinded investigators as previously described.^6^

### Genetic analyses

Genetic tests were non-systematically performed; their results were collected if applicable. Testing methods ranged from single-gene testing by Sanger sequencing or scanning methods such as denaturing high-performance liquid chromatography or high-resolution melting to gene panel testing with next-generation sequencing (NGS); both routine diagnosis testing and genetic testing for research purposes were collected. Next-generation sequencing panel testing included cardiac conduction genes (*SCN5A; LMNA; NKX2-5; HCN4; SCN1B; TRPM4*) and cardiomyopathy genes (see Supplementary methods). All variants were classified according to the American College of Medical Genetics and Genomics Guideline (ACMG) with standard terminology including benign, likely benign, variant of unknown significance (VUS), likely pathogenic (LP), and pathogenic (P). Only VUS/LP/P variants were reported.

### Statistical analysis

Categorical variables were presented as counts and percentages and continuous data as mean (±SD) or median [25th; 75th]. χ^2^ and Fisher’s exact tests were performed to compare groups. The Kaplan–Meier method estimator was used to assess the time of progression from incomplete to complete AVB (time between diagnosis and complete AVB or last follow-up) and the time from diagnosis to pacemaker implantation where appropriate. Cox proportional hazards regressions were used to analyse predictive factors, among baseline characteristics, of pacemaker implantation during follow-up. A two-sided *P*-value <0.05 was considered statistically significant. Data were analysed with the SAS packages (version 9.4, SAS Institute Inc., Cary, NC, USA).

## Results

A total of 385 unrelated children [192 boys, (49.9%)] were included in the study.

### Baseline clinical characteristics

#### Age at diagnosis

**Figure 1:**
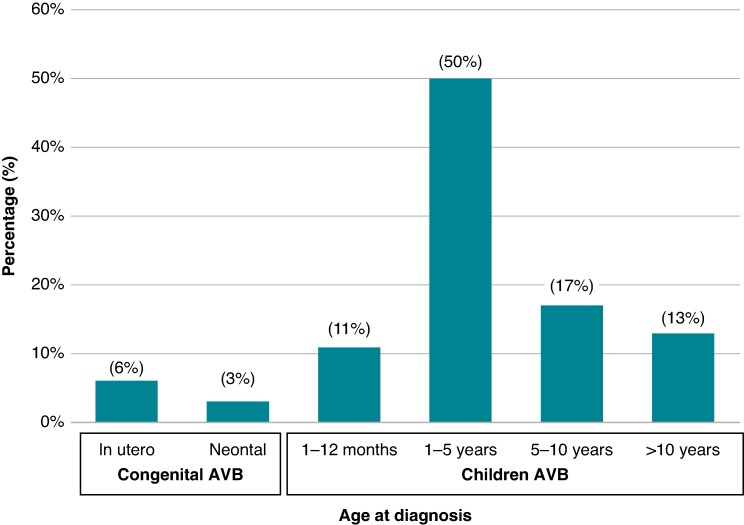
Age at the time of diagnosis of heart block. In 268 patients (69.6%), the diagnosis was made before 5 years of age. AVB, atrioventricular block.

The median age at diagnosis was 3.0 [1.0; 6.0] years ranging from *in utero* up to 18 years. Congenital AVB was diagnosed in 34 (8.8%) and childhood AVB in 351 (91.2%) patients. Congenital AVB was diagnosed *in utero* in 22 patients (5.7%), whilst 12 (3.1%) were diagnosed at birth or during the first month of life. Childhood AVB was diagnosed during the first year of life in 42 children (10.9%), between 1 and 5 years of age in 192 (49.9%), between 5 and 10 years of age in 66 (17.1%), and after 10 years of age in 51 (13.2%). The diagnosis was made before 5 years of age in 268 cases (69.6%) (*Figure 1*).

#### Clinical presentation at diagnosis

Most of the patients 314 (81.6%) were asymptomatic at diagnosis, the AVB being identified after the detection of bradycardia in 171 (58.6%), heart murmur in 90 (30.8%), both conditions in 31 (10.6%), and foetal bradycardia in 22 (7.1%). Atrioventricular block was diagnosed in 71 (18.4%) symptomatic children, who presented with syncope in 32 (45.1%), heart failure in 13 (18.3%), or fatigue in 25 (35.2%); in one child, the AVB was revealed by aborted cardiac arrest.

#### Foetal cases

In the 22 foetuses, AVB diagnosis was made at a median gestational age of 32.5 [28.0; 37.0] weeks, because of foetal bradycardia in 20 patients, hydrops fetalis in 1, and familial screening in 1 patient whose brother had been diagnosed at age 5 years with an isolated complete AVB. The ECG recorded at birth showed Type I second-degree AVB in 4 patients, Type II second-degree AVB in 6, and complete AVB in 12. Of the 22 foetal cases, 20 (90.9%) received a permanent pacemaker, implanted during the first week of life in 8 (36.4%) and during the first year of life in 17 (77.3%).

### Family history and parental ECG screening

A family history of unexplained syncope, sudden death, known cardiac conduction disorders (CCD), and/or pacemaker implantation before 50 years of age, was found in 2 (0.5%), 6 (1.6%), and 10 (2.6%), respectively. One consanguineous marriage between the parents of affected children was noticed. All parents were asymptomatic and in sinus rhythm except for one 56-year-old father with undetected complete AVB. Of the 204 parents who underwent an ECG screening, cardiac conduction abnormalities were found in 78 (38.2%), including long PR interval (14.2%), previously unrecognized complete AVB (0.5%), complete or incomplete right bundle branch block (30.9%), and complete or incomplete left bundle branch block (LBBB: 2.5%); 19 parents (9.3%) had prolonged corrected QT interval.

### Cardiac conduction disturbances

#### Conduction disturbances at diagnosis

Complete AVB was diagnosed in 263 (68.3%) patients, including 21 (8.0%) with congenital AVB and 242 (92.0%) with childhood AVB (*Table 1*). Incomplete AVB was diagnosed in 122 (31.7%), including 13 patients with congenital (10.7%) and 109 patients with childhood AVB (89.3%), ranging from first-degree AVB (33 patients, 27.0%), to Type I second-degree AVB (35 patients, 28.7%), or Type II second-degree AVB (54 patients, 44.3%). In the 26 (6.8%) patients whose QRS complex was wide, the intraventricular conduction morphology was complete LBBB in 9, left posterior fascicular block in 6, complete RBBB in 9, complete LBBB in 1, and a combination of RBBB and left anterior fascicular block in 3.

**Table 1:** Clinical characteristics at the time of diagnosis of incomplete and complete AVB

		Incomplete (*N* = 122)	Complete (*N* = 263)	*P*-value
Sex	Women	57 (46.7%)	136 (51.7%)	0.36
	Men	65 (53.3%)	127 (48.3%)	
Age at diagnosis	Min–max	[0.0; 17.0]	[0.0; 18.0]	**0**.**0109**
	Mean ± SD	3.6 ± 3.9	4.7 ± 4.7	
	Median [Q1; Q3]	2.0 [1.0; 6.0]	3.0 [0.0; 7.0]	
Congenital	*N* = 34	13 (10.7%)	21 (8.0%)	0.39
Childhood	*N* = 351	109 (89.3%)	242 (92.0%)	
Symptoms	No	101 (82.8%)	213 (81.0%)	0.67
	yes	21 (17.2%)	50 (19.0%)	
ECG at diagnosis	FC median [Q1; Q3]	70 [53.0; 100.0]	50 [45; 65]	**<0**.**0001**
	QRS Median [Q1; Q3]	70 [60.0; 80.0]	69 [60.0; 80.0]	0.31
	QTc Median [Q1; Q3]	409 [390; 425]	412 [400; 433]	0.20

Statistically significant *P*-value < 0.05 are marked in bold.

#### Progression of conduction disturbances

**Figure 2:**
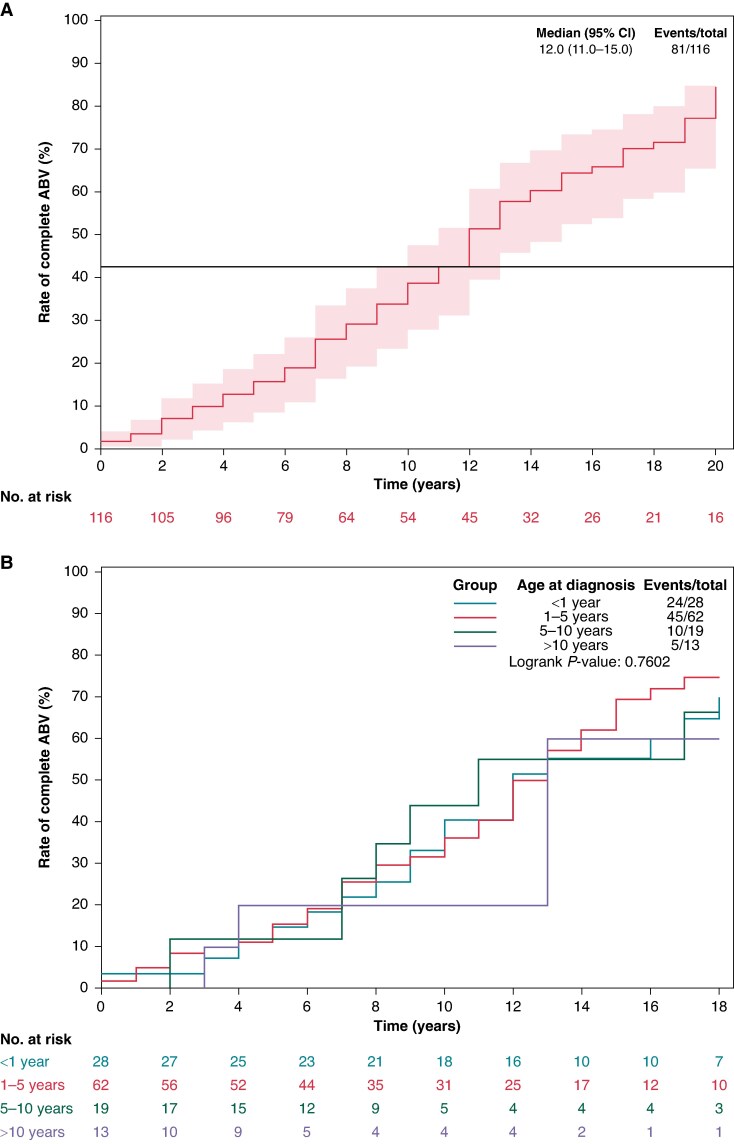
Risk of progression of incomplete AVB to complete AVB. (*A*) At 13 years, half of the children progressed from incomplete to complete AVB. (*B*) Curves according to age at diagnosis. AVB, atrioventricular block.

The median follow-up time was 10 [5; 17] years. Of the 263 patients who presented with a complete AVB at the time of diagnosis, 35 (13.3%) were paroxysmal complete AVB and 33 (94.3%) of those progressed to permanent complete AVB within a median of 10 years [4; 18]. Incomplete (first- or second-degree AVB) progressed to permanent, complete AVB within a median of 12 [7; 17] years in 84 of 122 patients (68.8%) (*Figure 2*).

### Pacemaker implantation

#### Age at pacemaker implantation

**Figure 3:**
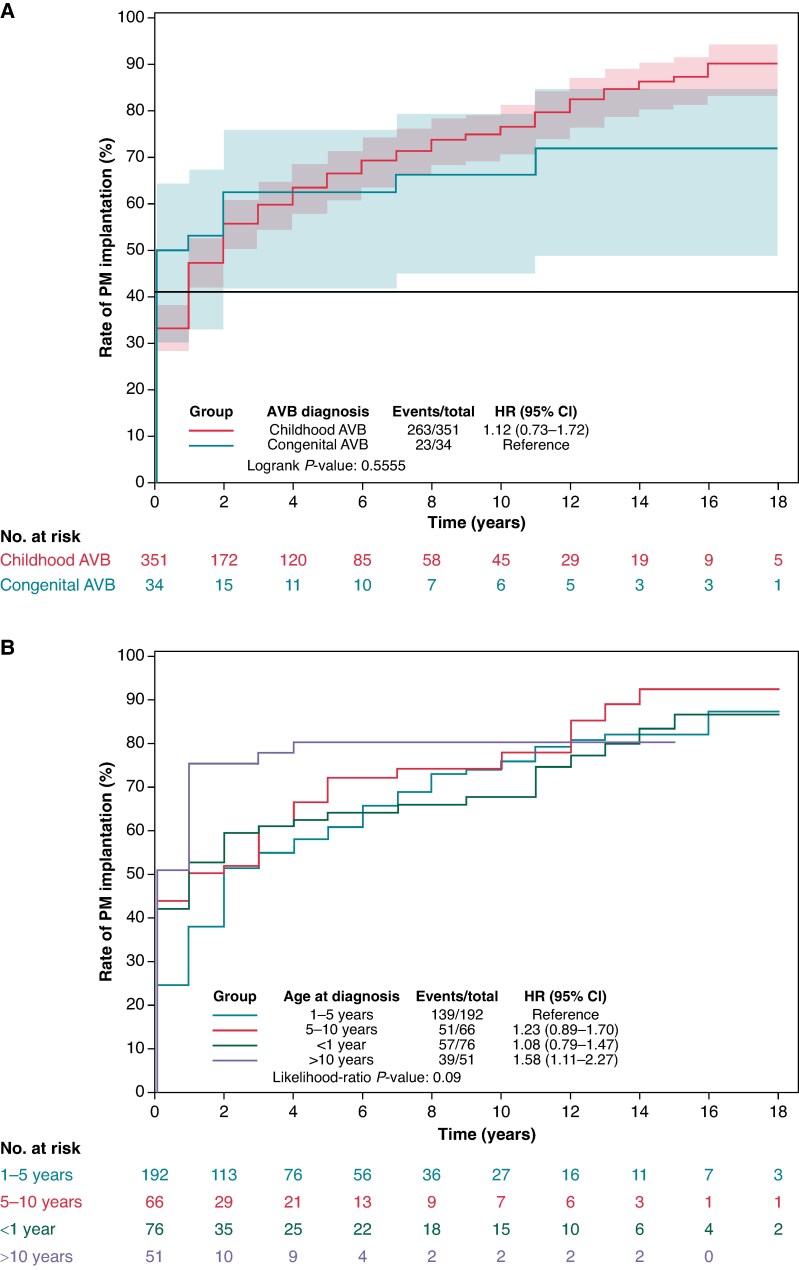
Rate of pacemaker implantation during long-term follow-up. (*A*) After a median follow-up of 10 years,^5–17^ 74.3% of the patients had received a permanent pacemaker, implanted in the first year of life in 7% and before 10 years of age in 77.6% of children. (*B*) Curves according to age at diagnosis.

During the follow-up, 286 (74.3%) received a permanent pacemaker, implanted during the first year of life in 39 (14%) and before 10 years of age in 172 (60%) children. The median age at pacemaker implantation was 5 years [2.0; 10.0] for the whole series; 1.2 month [1.2; 12] in the case of congenital AVB; and 6 years [3; 10] in the case of childhood AVB. The median time interval between AVB diagnosis and device implantation was 1 [1; 12] month in the case of congenital AVB and 12 [1; 36] months in the case of childhood AVB (log-rank *P* = 0.56) (*Figure 3*).

Overall, 99 (25.7%) children did not have a pacemaker implanted during follow-up. The median follow-up in this population was 6.0 [3.0; 11.0] years, 56 (56.6%) were boys, and only 11 children had a congenital AVB (11.1%). The median age at diagnosis was 3.0 [1.0; 6.0] years. At diagnosis, there were only 42 (42.5%) complete permanent or paroxysmal AVB. At last follow-up, 60 (60.6%) children had a complete AVB (see Supplementary material online, *Table S2*).

#### Conduction disorders and indications for cardiac pacing

At the time of pacemaker implantation, 262 (94.2%) had complete AVB with narrow QRS complex, including 19 (7.3%) congenital and 243 (92.7%) childhood AVB.

Of the 286 children implanted with a pacemaker, 203 (71.0%) were paced based on prophylactic indications, including a mean daytime heart rate <50 b.p.m. in children >1 year of age (118 patients, 58.7%), ventricular pauses longer than 3 RR intervals (63 patients, 31.0%), a heart rate <50 b.p.m. in infants (26 patients, 12.8%), a prolonged corrected QT interval (4 patients, 1.4%), and an escape rhythm with wide QRS complex (5 patients, 2.5%). Pacemaker was implanted for symptomatic bradycardia in 98 children (34.6%). Bradycardia-related symptoms included asthenia in 55 (19.2%), syncope in 40 (14.0%), exercise-induced dyspnoea in 23 (8.0%), heart failure in 12 (4.2%), and chest pain in 5 (1.7%).

#### Type and mode of cardiac pacing

Among 39 infants who received a permanent pacemaker before 1 year of age, epicardial leads were implanted in 36 (95%); two were implanted with endocardial leads, with a single-chamber device in 18 and a dual-chamber device in 21. Among 172 children between the ages of 1 and 10 years, 117 (69%) received epicardial leads and 53 (31%) received endocardial leads. Single-chamber pacemakers were implanted in 72 (42%), dual-chamber devices in 99 (58.4%), and a CRTP in 1. Among 75 children >10 years of age, 34 (46%) received epicardial leads, and 64 (88%) received a dual-chamber device.

#### Changes in the type of pacemaker implantation over time

**Figure 4:**
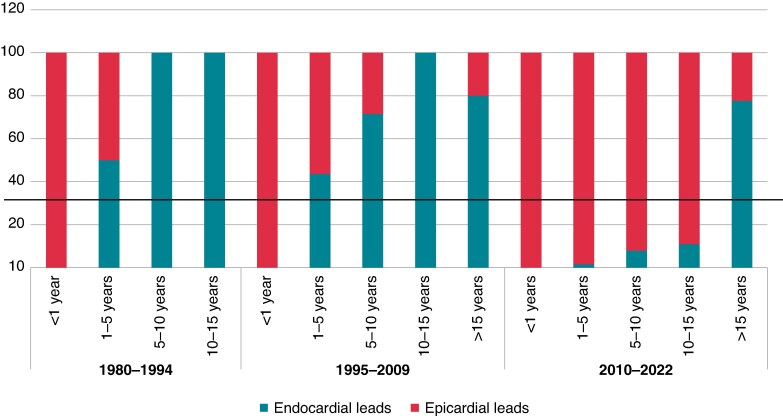
Type of pacemaker implantation according to age of implantation and studied period—endocardial or epicardial leads.

**Figure 5:**
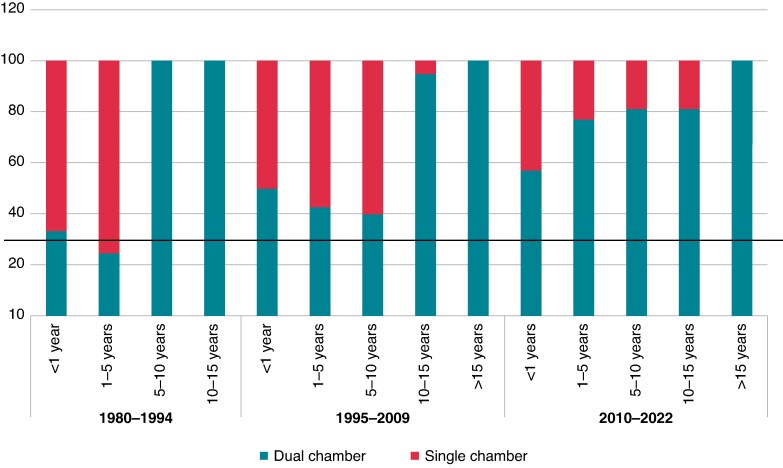
Type of first pacemaker implantation by age and time of studied period—single- or dual-chamber device.

We divided the study into three periods of time: before 1995, between 1995 and 2009, and after 2009. During the first period, 12 (70.6%) children <5 years of age were paced with a single-chamber device and one child >5 years received epicardial leads; during the second period, 30 (55.6%) children <5 years of age were paced with a single-chamber device and 14 (20.9%) children >5 years of age received epicardial leads; and during the third period, 15 (25%) children <5 years of age were paced with a single-chamber device and 69 (84.1%) children >5 years of age received epicardial leads (*Figures 4* and *5*, Supplementary material online, *Table S3*).

### Genetic analyses

Genetic screening was performed in 133 (34.5%) patients, consisting of a NGS gene panel testing in 114 (85.7%) patients and a single-gene testing in 19 patients. Of them, 11 (8.3%) had a variant in five genes: four in *SCN5A,* three in *TRPM4,* two in *TTN,* one in *LMNA,* and one in *PRKAG2*, including a pathogenic variant in seven (5.3%): five LP/P variants in *SCN5A* and *TRPM4* and two pathogenic variants in *TTN* (*Table 2*). Variants in *PRKAG2* and *LMNA* genes were classified as VUS. Two patients with VUS in *TRPM4* and *SCN5A* harboured a second VUS in *DSP* and *SNTA1.* The four *SCN5A* mutation-positive children had isolated AVB with no associated arrhythmias or structural heart abnormalities. Patients with a genetic variant had no more severe symptoms leading to AVB diagnosis than the rest of this cohort: bradycardia in eight, syncope in one, fatigue in one, cardiac murmur in one, and fatigue in one. Among them, seven (63.6%) required a pacemaker implantation. Only one child had a family history of CCD. The two patients with TTN pathogenic variants presented no sign of structural heart abnormalities, and we found no higher prevalence of ventricular arrhythmias in the patients with P/LP in SCN5A. Targeted testing of variants in parents of probands was performed in few families and identified a *de novo TRPM4* variant in proband N11.

**Table 2:** Clinical characteristics of patients with an identified genetic mutation

Patient	Genetic variant HGVS DNA and protein on transcript	ACMG classification	Sex	Age at diagnosis (years)	Symptoms	Conduction disorder	Pacemaker	Follow-up (years)	Complication	Familial history
N1	TRPM4 NM_017636.4 c.1127T > C p.Ile376Thr	P	M	0	Bradycardia	Complete AVB	1	22	Pacemaker infection	Grandmother had pacemaker
N2	PRKAG2 NM_016203.4 c.590C > G p.Pro197Arg	VUS	F	In utero	Bradycardia	Complete AVB	1	21	0	0
N3	TRPM4 NM_017636.4 c.2396G > C p.Arg799Pro; DSP NM_004415.4 c.2858T > C p.Leu953Pro	VUS VUS	M	2	Bradycardia	Type I second-degree AVB	0	7	0	0
N4	SCN5A NM_198056.2 c.4747C > T p.Arg1583Cys	LP	M	12	Bradycardia	First-degree AVB	0	2	0	0
N5	TTN NM_001256850.1 c.66055C > T p.Arg22019*	P	F	0.9	Syncope	Type I second-degree AVB	1	22	0	0
N6	TTN NM_001256850.1 c.57154_57157del p.Asp19052Metfs*62	P	F	15	Dyspnoea	Complete AVB	1	3	0	0
N7	LMNA NM_170707.3 c.1580G > A p.Arg527His	VUS	M	7	Bradycardia	Type I second-degree AVB	1	7	0	0
N8	SCN5A NM_198056.2 c.1100G > A p.Arg367His	P	M	1	Bradycardia and cardiac murmur	Type I second-degree AVB	1	13	Pacemaker infection	Grand mother and aunt had pacemaker
N9	SCN5A NM_198056.2 c.3835G > A p.Val1279Ile; SNTA1 NM_003098.2 c.992G > T p.Arg331Leu	VUS VUS	M	6	Cardiac murmur	Type I second-degree AVB	0	2	0	0
N10	SCN5A NM_198056.2 c.5417_5420del p.Thr1806Serfs*27	P	F	7	Bradycardia	First-degree AVB	0	3	0	0
N11	TRPM4 NM_017636.4 c.2030C > T p.Thr677Ile (*de novo*)	P	M	0.3	Bradycardia	Complete AVB	1	30	0	0

All variants reported were heterozygous.

LP, likely pathogenic; P, pathogenic; VUS, variant of uncertain significance.

### Clinical outcomes

The follow-up was >10 years in 183 patients (47.4%) and >15 years in 102 (26.5%) patients. After a median follow-up time of the entire study population of 10 [5; 17] years, no patient died or developed DCM, and 271/286 (94.8%) remained free from pacemaker-related complications at last visit. Of the children who experienced pacemaker-related complications, 35 (12.2%) had major ones, with cardiac tamponade in 1, lead rupture/dysfunction in 31, and pacemaker lead endocarditis in 3 after 4, 6, and 11 years of cardiac pacing, respectively. Of the 286 children who were implanted with a pacemaker, 146 (51%) required at least one reintervention. Among the 187 patients implanted with an epicardial pacemaker, 86 (59%) had a reintervention after a median follow-up of 8.7 [4; 12] years, 64 (34.2%) for battery replacement and 22 (12%) for an unplanned reintervention (21 lead fractures or dysfunctions and 1 lead infection). Among the 95 patients who had an endocardial pacemaker, 59 (41%) had a reintervention after a median follow-up of 13.7 [5; 17] years, 46 (46%) for battery replacement and 13 (13%) for an unplanned reintervention (10 lead fractures or dysfunctions, 2 infections, and 1 due to lead placement anomaly).

## Discussion

This study reports the clinical evaluation and follow-up of the largest paediatric population of non-immune, isolated AVB individuals reported to date. Our major finding was highly favourable long-term outcomes of these patients presenting with this rare form of paediatric idiopathic AVB.

It has been proposed that late-onset DCM in congenital AVB patients may be a sequela of *in utero* autoimmune myocarditis or due to its post-natal reactivation.^8^ We previously showed that immune and non-immune AVB patients have different clinical outcomes, non-immune ones being diagnosed and paced later in life and having a better prognosis than the immune ones, because of a high neonatal mortality rate and a high risk of DCM in immune-isolated AVB patients.^8^ We later reported a favourable long-term prognosis of non-immune, isolated AVB, regardless of the patient’s age at the time of diagnosis, on a multicentre series of 141 patients.^6^ Our results extend this observation to a larger series with an extended follow-up, as no death and no DCM were observed in 385 patients over a median follow-up of 10 years.

Our results contrast with a recent Danish retrospective, case–control observational study, which suggested that young patients paced for AVB of unknown aetiology had a 3.8-fold higher rate of death, hospitalization for heart failure, ventricular tachyarrhythmias, or aborted sudden death, compared with an age- and sex-matched control population.^7^ These data raise questions about what exactly is meant by ‘unknown aetiology’. The 50.3% rate of AVB of unknown aetiology emphasizes the lack of thorough case investigations in the Danish cohort.^9^ The poor outcome observed in their patients may be due to an unknown underlying disease, which has been insufficiently investigated. Diagnostic work-up was not standardized, and both maternal immune status and genetic testing were lacking in most patients. Particularly, the low 0.6% rate of genetic AVB in the Danish cohort does not reflect the true prevalence of hereditary AVB, as *SCN5A* mutations account for 5% of CCD in the young.^10^ This is due to limited genetic testing in the studied population.^11^ Nevertheless, genetic testing should be considered in patients with early-onset (age <50 years) CCD,^10^ as genetic variants in multiple genes have been described. Here, genetic mutations with a strong impact on patients’ outcomes such as *LMNA* mutations or *SCN5A* mutations possibly in the setting of cardiac sodium channelopathy overlap syndrome^12^ may have been missed.

The absence of antibody in non-immune, isolated AVB suggested a different pathologic mechanism than autoimmunity. In a previous study, we showed that 70% of those with incomplete AVB at the time of diagnosis progressed to a complete one, suggesting a genetic predisposition to a progressive process of the specialized conduction tissue.^6^ Given that an increasing number of genes have been found to be responsible for hereditary progressive cardiac conduction defect in adults^13–16^ and that several common variants modulate heart rate, PR interval, and QRS complex durations,^17–19^ we hypothesized that idiopathic AVB in the young may be a heritable disease. Electrocardiogram screening in parents of children with idiopathic AVB as compared to matched healthy control subjects revealed a high prevalence of conduction abnormalities as well as a high estimated heritability for isolated conduction disturbances, which supported the hypothesis of an inheritable trait in congenital and childhood non-immune, isolated AVB.^20^ Genetic investigations in that cohort later confirmed culprit mutations in *SCN5A*,^12,20^  *TRPM4*^21^ and in the *Connexin 40*^22^ and *Connexin 45* genes.^23^ However, the rate of variants identified remains low, which shows that our genetic knowledge in this field remains limited. Our study findings are unaligned with a recent small Italian monocentre retrospective series of 39 young adults with CCD of unknown origin, in which a comprehensive cardiomyopathy and arrhythmia gene panel had an overall detection rate of 38%.^24^ The proportion of genetic findings is likely overestimated in their study, given that an underlying cardiomyopathy (DCM or LV non-compaction) and sick sinus syndrome were reported in 13 and 33% of their patients, respectively. It is also unclear whether their diagnostic work-up would have identified an underlying neuromuscular disorder or metabolic disease, and no follow-up data were provided. Moreover, classical genetic approaches in this field may not be the most appropriate, and it is probably necessary to use new approaches, such as the use of trios to identify other genetic mechanisms.

In our study, no patient developed DCM or required biventricular pacing after a median follow-up of 10 years. Choosing the optimal pacing site for chronic ventricular pacing has been identified to be of major impact in preserving ventricular function. Pacing-induced cardiomyopathy resulting from the detrimental effects of right ventricular apical pacing in the young has well been described.^25,26^ Pacing leads were mostly placed on the LV apex, as LV pacing preserves LV systolic function and synchrony in neonates and infants with congenital complete AVB over time^27^ and is now accepted as the preferred site for epicardial pacing.^28,29^ Of note, we report a shift in cardiac pacing modalities along the study period. In the last decade as compared to earlier era, there has been an increase in the use of epicardial leads in children aged 5–15 years requiring pacing. In our experience, the rate of unplanned reinterventions was similar between epicardial and endocardial pacing systems, although the follow-up of children who received an epicardial pacemaker was shorter. Children might be more prone to complications because of their active lifestyle, higher frequency of traumatic events, and infections that affect the pacing system. Within those limitations, there is a consensus that the smallest infants are best served with epicardial pacing systems, with a cut-off weight around 15–20 kg.^26^ Epicardial leads are more likely to fracture and are prone to exit block, and implantation requires a major operation that is accompanied by the inherent risks and need for perioperative support.^30^ On the contrary, endocardial systems may carry a significant risk of venous thrombosis in young patients, which can result in loss of venous access in the future, leading to a more complicated lead revision later in the patient’s life.^31^ Up to a 19% transvenous lead-related failure rate has been reported by others who choose the endocardial approach. Extraction of abandoned transvenous leads in the paediatric population is also problematic, and optimal lead management remains to be defined. This should explain the observed trend with an increase in epicardial pacemaker implants in subjects over 5 years of age over time.^30^

### Study limitations

First, the retrospective data collection over 42 years at multiple centres may have underestimated the incidence of adverse events during follow-up. However, the follow-up of children with AVB (with or without pacemaker implanted) requires at least 1 to 2 medical consultations annually, which limits the likelihood of underreported clinically significant events. The retrospective nature of this study represents a limitation as genetic testing has evolved, so that some cardiac conduction system genes, channelopathy genes, and cardiomyopathy genes may have not been tested. As the review of medical records was not exhaustive at all participating centres, the prevalence of idiopathic AVB may have been underestimated.

## Conclusions

In this large paediatric cohort of non-immune, isolated AVB of unknown cause, the long-term outcome was favourable with no death, no DCM, and relatively few pacemaker-related complications. Nearly 70% of patients with incomplete AVB progressed to a complete one, suggesting a genetic predisposition to a progressive process of the cardiac conduction system. Among patients with a genetic testing, 5.3% had an identified pathogenic variant. Genetic testing using an arrhythmia and cardiomyopathy genes panel should be considered in every infant or child with cardiac conduction system disease and whose mothers are anti-Ro/SSA negative, with cascade testing whether they are positive. Further investigations are needed to determine (a) the very long-term evolution in adulthood and (b) the aetiology and pathophysiology of this rare type of conduction disorder.

## Supplementary Material

euaf040_Supplementary_Data

## Data Availability

In accordance with the Transparency and Openness Promotion Guidelines, the data that support the findings of this study are available from the corresponding author on reasonable request.

## Author information

**The Congenital AVB study group:** Florence Mycinski (Nantes Université, France), Victor Waldmann (Hôpital Necker Enfants Malades, France), Florence Kyndt (Nantes Université, France), Béatrice Guyomarch (Nantes Université, France), Alice Maltret (Université Paris Sud, France), Marie Wilkin (Aix-Marseille Université, France), Caroline Ovaert (Aix-Marseille Université, France), Guy Waksmann (Hôpital La Louvière, France), Jean-Benoit Thambo (Université de Bordeaux, France), Solène Prigent, (CHU Nantes, France), Claire Galoin-Bertail (CHU Lyon, France), Gilles Bosser (CHU Nancy, France), Céline Grunenwald (CHU Nantes, France), Grégoire De La Villeon (CHU Montpellier, France), Bruno Lefort (CHU Tours, France), Sylvie Falcon-Eicher (CHU Dijon, France), Matthias Lachaud (CHU Grenoble, France), François Godart (CHU Lille, France), Raphaël P. Martins (CHU Rennes, France), Claire Dauphin (CHU Clermont-Ferrand, France), Hervé Joly (Clinique du Val d'Ouest, France), Isabelle Durand (CHU Rouen, France), Clément Karsenty (CHU Toulouse, France), Guillaume Deverrière (CHU Brest, France), Pierre Mauran (American Memorial Hospital, France), Bérangère Urbina-Hiel (CHU Amiens, France), Cécile Pascal (Hôpital du Confluent, France), Adeline Basquin (Cabinet de la Cochardière, France), Laure Ponthier (CHU Limoges, France), Karim Jamal-Bey (CHU La Réunion, France), Jean-Marc Sellal (Université de Lorraine, France), Paul Padovani (Nantes Université, France), Naychi Lwin (Nantes Université, France), Solène Prigent (Nantes Université, France), Oscar Werner (Nantes Université, France), Julien Barc (Nantes Université, France), Jean-Jacques Schott (Nantes Université, France), Damien Bonnet (Université Paris Cité, France), Vincent Probst (Nantes Université, France), Alban-Elouen Baruteau (Nantes Université, France).
