# Supplementary material for: Late outcomes of congenital and childhood non-immune, isolated atrioventricular block: a French nationwide retrospective cohort study
Source: Europace. 2025 Mar 11;27(3):euaf040. doi: 10.1093/europace/euaf040 (PMC11919819; doi:10.1093/europace/euaf040)
Supplement: euaf040_Supplementary_Data [file euaf040_supplementary_data.pdf]

SUPPLEMENTAL MATERIALS

SUPPLEMENTARY METHODS

Definitions. AVB was classified as congenital if it was diagnosed *in utero*, at birth or during the first month of life, vs. childhood AVB if diagnosed between the first month and 18 years old (1). Atrioventricular and intraventricular conduction abnormalities were classified according to age using the definitions of international practice guidelines (**Supplementary Table S1**) (2,3-5). Dilated cardiomyopathy (DCM) was defined as left ventricular (LV) dilatation with a LV end-diastolic diameter greater than two standard deviations (Z-scores >+2), and LV systolic dysfunction with a shortening fraction <25% on two-dimensional transthoracic echocardiogram (6).

Genetic analyses. Next Generation Sequencing panels included both cardiac conduction genes *(SCN5A; LMNA; NKX2,5; HCN4; SCN1B; TRPM4)* and cardiomyopathy genes (*ABCC9*, *ACTC1*, *ACTN2*, *ANKRD1*, *BAG3*, *CALR3*, *CAV3*, *CSRP3*, *DES*, *DSC2*, *DSG2*, *DSP*, *DTNA*, *EMD*, *GAA*, *GATA4*, *GJA1*, *GJA5*, *GJC1*, *GLA*, *HCN4*, *JPH2*, *JUP*, *LAMP2*, *LDB3*, *LMNA*, *MYBPC3*, *MYH6*, *MYH7*, *MYL2*, *MYL3*, *MYLK2*, *MYOZ2*, *MYPN*, *NEXN*, *NKX2-5*, *PKP2*, *PLN*, *PRKAG2*, *RBM20*, *RYR2*, *SCN5A*, *SGCD*, *SLC22A5*, *TAZ*, *TBX5*, *TCAP*, *TMEM43*, *TNNC1*, *TNNI3*, *TNNI3K*, *TNNT2*, *TPM1*, *TTN*, *VCL*).

SUPPLEMENTAL TABLES

Supplementary Table S1: Considered cut-off values for definition of cardiac conduction abnormalities according to age

	**< 4 years**	**Between 4-16 years**	**> 16 years**
**1st degree AV block (PR,ms)**	≥ 160	≥ 180	≥ 200
**Incomplete RBBB (QRS,ms) □**	80 ≤ QRS ≤ 90	90 ≤ QRS ≤ 100	110 ≤ QRS ≤ 120
**Complete RBBB (QRS,ms) □**	≥ 90	≥100	≥120
**Incomplete LBBB (QRS,ms) #**	80 ≤ QRS ≤ 90	90 ≤ QRS ≤ 100	110 ≤ QRS ≤ 120
**Complete LBBB (QRS,ms) ##**	≥ 90	≥100	≥120
**Left anterior fascicular block (QRS,ms)**	< 120 *	< 120 *	< 120 *
**Left posterior fascicular block (QRS,ms)**	< 120 **	< 120 **	< 120 **
**Right axis deviation (QRS axis,°)**	+180° and beyond	+140° and beyond	+90° and beyond
**Left axis deviation (QRS axis,°)**	-30° and beyond	-30° and beyond	-30° and beyond

BBB: bundle branch block

□ and rsr', rsR' or rSR' in leads V1 or V2

# and absent q wave in leads I, V5 and V6; and R peak time > 60 ms in leads V5 and V6 but normal in leads V1, V2 and V3.

## and broad notched or slurred R wave in leads I, aVL, V5 and V6, eventually associated with a RS pattern in V5 and V6; and absent q wave in leads I, V5 and V6; and R peak time > 60 ms in leads V5 and V6 but normal in leads V1, V2 and V3.

**and frontal plane axis between -45° and -9°°; and qR pattern in leads aVL; and R peak time > 45ms in aVL*

***and frontal plane axis between 100° and 180°; and rS pattern in leads I and aVL; and qR pattern in leads III and aVF*

Adapted from :

Baruteau AE, Kyndt F, Behr ER, Vink AS, Lachaud M, Joong A, et al. SCN5A mutations in 442 neonates and children: genotype-phenotype correlation and identification of higher-risk subgroups. Eur Heart J. 2018;39:2879-2887. doi: 10.1093/eurheartj/ehy412.PMID: 30059973

Supplementary Table S2: Characteristics of the 99 patients who did not receive a permanent cardiac pacemaker over the follow-up.

Characteristics		N= 99
**Sex**	***Women***	43 (43.4%)
	Men	56 (56.6%)
**Age at diagnosis**	***Min-Max***	[0.0;18]
	Mean+/- sd	4.1+/-4.2
	Median [Q1; Q3]	3.0[1.0;6.0]
**Congenital**		11 (11.1%)
**Childhood**		88 (88.9%)
**Time of follow-up (years)**	***Min-Max***	[0.0;35.0]
	Mean+/- sd	7.5+/-6.4
	Median [Q1; Q3]	6.0[3.0;11.0]
**Type of AVB at diagnosis**	***Complete***	42 (42.4%)
	Incomplete	57 (57.6%)
**Type of AVB at last follow up**	***Complete***	60 (60.6%)
	Incomplete	39 (39.4%)

Supplementary Table S3: The different types of pacemakers implanted according to age and period of implantation

	Period (years)													
	1980-1994				1995-2009					2010-2022				
	<1 year	1-5 years	5-10 years	10-15 years	<1 year	1-5 years	5-10 years	10-15 years	>15 years	<1 year	1-5 years	5-10 years	10-15 years	>15 years
**Dual chamber (%)**	33.3	25	100	100	50	42.9	40	95	100	57.1	77.0	81.1	81.5	100
**Mono chamber (%)**	66.7	75	0	0	50	57.1	60	5	0	42.9	23.0	18.9	18.5	0

	Period (years)													
	1980-1994				1995-2009					2010-2022				
	<1 year	1-5 years	5-10 years	10-15 years	<1 year	1-5 years	5-10 years	10-15 years	>15 years	<1 year	1-5 years	5-10 years	10-15 years	>15 years
**Endocardial leads (%)**	0	50	100	100	0	43.6	71.4	100	80	0	1.6	8.1	10.7	77.8
**Epicardial leads (%)**	100	50	0	0	100	56.4	28.6	0	20	100	98.4	91.9	89.3	22.2

SUPPLEMENTARY REFERENCES

1. Saxena A, Izmirly PM, Mendez B, Buyon JP, Friedman DM. Prevention and treatment in utero of autoimmune-associated congenital heart block. Cardiol Rev. 2014;22:263-7.
2. Baruteau AE, Fouchard S, Behaghel A, Mabo P, Villain E, Thambo JB, et al. Characteristics and long-term outcome of non-immune isolated atrioventricular block diagnosed in utero or early childhood: a multicentre study. Eur Heart J. 2012;33:622-9.
3. Schwartz PJ, Garson A, Paul T, Stramba-Badiale M, Vetter VL, Wren C, et al. Guidelines for the interpretation of the neonatal electrocardiogram. A task force of the European Society of Cardiology. Eur Heart J. 2002;23:1329-44.
4. Surawicz B, Childers R, Deal BJ, Gettes LS, Bailey JJ, Gorgels A, et al. AHA/ACCF/HRS recommendations for the standardization and interpretation of the electrocardiogram: part III: intraventricular conduction disturbances: a scientific statement from the American Heart Association Electrocardiography and Arrhythmias Committee, Council on Clinical Cardiology; the American College of Cardiology Foundation; and the Heart Rhythm Society. Endorsed by the International Society for Computerized Electrocardiology. J Am Coll Cardiol. 2009;53:976-81.
5. Rijnbeek PR, Witsenburg M, Schrama E, Hess J, Kors JA. New normal limits for the paediatric electrocardiogram. Eur Heart J. 2001;22:702-11.
6. Lipshultz SE, Law YM, Asante-Korang A, Austin ED, Dipchand AI, Everitt MD, et al. Cardiomyopathy in Children: Classification and Diagnosis: A Scientific Statement From the American Heart Association. Circulation. 2019;140:e9-68.
